# Systemic and central nervous system neuroinflammatory signatures of neuropsychiatric symptoms and related cognitive decline in older people

**DOI:** 10.1186/s12974-022-02473-3

**Published:** 2022-05-28

**Authors:** Christopher Clark, Jonas Richiardi, Bénédicte Maréchal, Gene L. Bowman, Loïc Dayon, Julius Popp

**Affiliations:** 1 Institute for Regenerative Medicine, University of Zürich, Wagistrasse 12, 8952 Schlieren, Switzerland; 2 Department of Radiology, Lausanne University Hospital and University of Lausanne, 1011 Lausanne, Switzerland; 3 Advanced Clinical Imaging Technologies Group, Siemens Healthcare Switzerland, 1015 Lausanne, Switzerland; 4 Nestlé Institute of Health Sciences, Nestlé Research, EPFL Innovation Park, Bâtiment H, 1015 Lausanne, Switzerland; 5 Nestlé Institute of Food Safety & Analytical Sciences, Nestlé Research, EPFL Innovation Park, Bâtiment H, CH-1015 Lausanne, Switzerland; 6 Institut Des Sciences et Ingénierie Chimiques, Ecole Polytechnique Fédérale de Lausanne, CH-1015, Lausanne, Switzerland; 7 Centre Hospitalier Universitaire Vaudois, Rue du Bugnon 46, 1011 Lausanne, Switzerland; 8 Department of Geriatric Psychiatry, Centre for Gerontopsychiatric Medicine, University Hospital of Psychiatry Zürich, Minervastrasse 145, P.O. Box 341, 8032 Zurich, Switzerland; 9 Department of Neurology, NIA-Layton Aging and Alzheimer’s Disease Research Center, Oregon Health & Science University, Portland, Oregon USA; 10 Helfgott Research Institute, National University of Natural Medicine, Portland, Oregon USA; 11 Department of Psychiatry, Psychotherapy and Psychosomatics, Psychiatric Hospital of the University of Zurich, Lengstrasse 31, Zürich, Switzerland

**Keywords:** Neuroinflammation, Neuropsychiatric symptoms, Alzheimer’s disease, biomarkers, Cerebrospinal fluid, Serum, Cognitive decline

## Abstract

**Background:**

Neuroinflammation may contribute to psychiatric symptoms in older people, in particular in the context of Alzheimer’s disease (AD). We sought to identify systemic and central nervous system (CNS) inflammatory alterations associated with neuropsychiatric symptoms (NPS); and to investigate their relationships with AD pathology and clinical disease progression.

**Methods:**

We quantified a panel of 38 neuroinflammation and vascular injury markers in paired serum and cerebrospinal fluid (CSF) samples in a cohort of cognitively normal and impaired older subjects. We performed neuropsychiatric and cognitive evaluations and measured CSF biomarkers of AD pathology. Multivariate analysis determined serum and CSF neuroinflammatory alterations associated with NPS, considering cognitive status, AD pathology, and cognitive decline at follow-up visits.

**Results:**

NPS were associated with distinct inflammatory profiles in serum, involving eotaxin-3, interleukin (IL)-6 and C-reactive protein (CRP); and in CSF, including soluble intracellular cell adhesion molecule-1 (sICAM-1), IL-8, 10-kDa interferon-γ-induced protein, and CRP. AD pathology interacted with CSF sICAM-1 in association with NPS. Presenting NPS was associated with subsequent cognitive decline which was mediated by CSF sICAM-1.

**Conclusions:**

Distinct systemic and CNS inflammatory processes are involved in the pathophysiology of NPS in older people. Neuroinflammation may explain the link between NPS and more rapid clinical disease progression.

**Supplementary Information:**

The online version contains supplementary material available at 10.1186/s12974-022-02473-3.

## Background

Neuropsychiatric symptoms (NPS) are frequent in older people and may represent the clinical manifestation of a variety of acute and chronic cerebral pathologies. NPS are particularly common among patients with Alzheimer’s disease (AD) [1, 2], and may precede the clinical stages of dementia [3, 4] and mild cognitive impairment (MCI) [5]. In the context of AD, early occurring NPS increase clinical disease severity and caregiver burden, and have been associated with more rapid cognitive and functional decline [2, 5–7].

Increased neuroinflammatory activity and altered cytokine levels in the CNS have been related to psychiatric and behavioral syndromes. The activation of the central innate immune response by cytokines initiates a behavioral response known as sickness behavior [8] which is characterized by the co-occurrence of anxiety, depression, and apathy. In animal models, this increase in cytokine levels is also associated with increased neurodegeneration [9–11]. In humans, chronic inflammatory diseases and altered cytokine levels are associated with NPS such as depression, anxiety and apathy [12–15]. In older people, both systemic and CNS inflammation have been associated with NPS in general [16], and in relation to neurocognitive disorders in particular [17–20]. In the context of AD, neuroinflammatory markers present in peripheral blood may indicate cerebral pathology or systemic inflammation relevant for the core AD processes [19, 21] and be associated with disease severity [22]. However, the specific inflammatory markers associated with NPS and their possible contribution to the clinical course remain largely unknown.

In this study we analyzed a large panel of neuroinflammatory markers at systemic and CNS levels, and hypothesized that specific circulating blood and CSF neuroinflammatory marker profiles are associated with the occurrence of NPS in general and of individual syndromes such as depression, anxiety and apathy in older subjects with normal cognition or with beginning cognitive decline. A further objective was to determine how these inflammatory profiles change depending on the presence of cerebral AD pathology and/or cognitive impairment. Finally, we verified if the identified inflammatory signatures were associated with clinical disease progression at follow-up visits.

## Methods

### Study population

We enrolled 87 community dwelling individuals, aged 55 or older, from a brain aging study conducted in the Department of Psychiatry and the Department of Clinical Neurosciences, University Hospital of Lausanne, Switzerland. Cognitively impaired participants were recruited among memory clinic outpatients and displayed no major psychiatric or neurological disorders, nor substance abuse or severe or unstable physical illness that may contribute to cognitive impairment, including history or clinical signs of inflammation. All subjects in this group had clinical diagnoses of either MCI or mild dementia, and a Clinical Dementia Rating (CDR [23]) score ≥ 0.5  and displayed memory impairment and/or impairment in other cognitive domains such as executive tasks or language skills [24]. Cognitively healthy participants were recruited through journal announcements or word of mouth and had no history, symptoms or signs of relevant psychiatric or neurological disease and no cognitive impairment. An overall clinical, neurological and comprehensive neuropsychological assessment and the administration of informant questionnaires was performed for all participants as previously described [19]. Additional clinical and neuropsychological follow-up evaluations were performed at 18 and 36 months using the same methods and tests whenever possible.

### Study procedures

#### Neuropsychological cognitive and neuropsychiatric assessments

The neuropsychological assessment and the administration of informant questionnaires to the participant’s proxy was previously described [19]. Briefly, cognitive performance was assessed with the Clinical Dementia Rating (CDR), CDR Sum of Boxes (CDR-SoB) and Mini-Mental State Examination (MMSE). The neuropsychological assessment also included the Buschke Double Memory Test [25], the Stroop Test [26], and the Trail Making Tests A and B [27]. Functional assessment included the activities of daily living (ADL) and instrumental ADL (IADL) tests [28]. All participants with CDR ≥ 0.5 were considered cognitively impaired and those with CDR = 0 as cognitively healthy. The Neuropsychiatric Inventory questionnaire (NPI-Q) [29] was administered to assess neuropsychiatric symptoms. Twelve categories, ten behavioral and two neurovegetative (Night-time behavior and Appetite/Eating), were scored for their severity ranging from 0 to 3. Total NPI-Q score was obtained by adding the 12 scores. Participants were considered to have NPS if the total NPI-Q score was > 0.

#### Biochemical sample collection and handling

Lumbar and venous punctures were conducted during the same visit between 8:30 and 10 a.m. after an overnight fast yielding 10–12 ml of CSF and 40 ml of blood, respectively, were performed, spun down at 4 °C, immediately aliquoted, and snap frozen at − 80 °C until assayed [19]. Study personnel blinded to clinical data performed biochemical and genetic analyses.

#### Biochemical measures

The CSF albumin index (Qalb) as a marker of blood–brain barrier (BBB) function along with the Apolipoprotein E (*APOE*) genotype were determined as previously described [30].

#### Cerebrospinal fluid AD biomarkers

CSF beta-amyloid 1–42 (Aβ1–42), total-tau (Tau), and tau phosphorylated at threonine 181 (pTau181) concentrations were measured using commercially available ELISA kits (Fujirebio, Gent, Belgium). A pTau181/Aβ_1–42_ ratio > 0.0779 was defined as an AD CSF profile as previously described [19]. Briefly, this value was determined using center data obtained from the study cohort and was the fitted value that that maximized the Youden index in a ROC analysis predicting CDR category [24].

#### Inflammation marker measurements

Quantitative analysis of 38 neuroinflammatory biomarkers in both CSF and serum was achieved using a sandwich immunoassay (Meso Scale Diagnostics, Rockville, USA) as previously described [30]. Biomarkers with more than 5% missing data or below level of quantification were filtered out, resulting in a selection of 28 analytes. These included basic fibroblast growth factor, C-reactive protein (CRP), eotaxin-1, eotaxin-3, interferon-γ (IFN-γ), interleukin-12, interleukin-15 (IL-15), interleukin-16 (IL-16), interleukin-6 (IL-6), interleukin-7, interleukin-8 (IL-8), 10-kDa interferon-γ-induced protein (IP-10), monocyte chemoattractant protein 1 (MCP-1), monocyte chemoattractant protein 4 (MCP-4), macrophage-derived chemokine (MDC), macrophage inflammatory protein 1α (MIP-1α), macrophage inflammatory protein 1β, phosphorylated insulin-like growth factor-1 receptor, serum amyloid A, soluble fms-like tyrosine kinase-1 (sFLT-1), soluble intercellular adhesion molecule-1 (sICAM-1), circulating vascular cell adhesion molecule-1 (sVCAM-1), thymus and activation-regulated chemokine (TARC), angiopoietin-1 receptor (TIE-2), tumor necrosis factor-α, vascular endothelial growth factor (VEGF), vascular endothelial growth factor C and vascular endothelial growth factor D precursor (VEGF-D). Prior to analysis, outliers within all measured markers exceeding the cutoff value of mean ± 3 × SD were replaced by the cutoff value. Neuroinflammatory marker measurements were also log_*n*_-transformed prior to correlation and regression analyses to approach Gaussian distribution. Normal distribution was subsequently assessed using the Kolmogorov–Smirnov test that identified 6 neuroinflammatory biomarkers in CSF (IL-7, MCP-4, MDC, MIP-1α, SAA, TARC) and 5 in serum (eotaxin-3, IL-6, MIP-1α, TIE-2, VEGF-C) not normally distributed.

### Statistical and analytical approaches

Descriptive statistics for the cohort were performed using *t*-tests comparing participants with and without NPS for continuous variables and Chi-square tests for categorical variables. Correlations between NPI-Q total score and neuroinflammatory markers were assessed with Spearman’s rho. Independence of variables used in regression models was tested with variance inflation factor (VIF). No variable entered in these models had VIF above 7, with a majority below 3, demonstrating absence of multicollinearity and allowing regression models to be used. Benjamini–Hochberg correction of *P*-value for multiple testing was applied for all analyses using a false-discovery rate of 0.25. Statistical data analysis was performed with IBM SPSS Statistics software version 25. All statistical models performed in this study were verified for possible overfitting using the Hosmer–Lemeshow test for goodness-of-fit. Models with a Hosmer–Lemeshow Chi-squared value yielding a *P*-value > 0.05 were rejected and the previous iteration was considered instead.

### Association between NPS and neuroinflammation markers

In order to identify neuroinflammatory marker combinations in CSF or serum associated with NPS, we used binary regression models with NPI-Q > 0 or NPI-Q = 0 as dependent variable while entering either all CSF or all serum markers in the models. We explored the effect of the following confounders: cognitive status (CDR = 0 or CDR > 0), CSF AD biomarker profiles (pTau181/Aβ_1–42_ ratio) and BBB function (Qalb) by entering them into the model before considering inflammatory marker concentrations. All models used a forward selection method based on the significance of the score statistic to select the smallest possible number of molecules associated with the occurrence of NPS in each case.

### Associations of single neuropsychiatric syndromes with neuroinflammation markers

To determine inflammatory marker signatures associated with the presence of individual syndromes (NPI-Q > 0 for each individual category) linked to sickness behavior and associated with neuroinflammation in the literature, i.e., depression, apathy and anxiety, we used binary logistic regression models corrected for cognitive status (CDR = 0 or CDR > 0) as above. These models used a forward selection method based on the significance of the score statistic to select the smallest number of molecules associated with each individual symptom.

### Interactions between cognitive status, AD pathology, and the associations of inflammatory markers with NPS

The interactions between an AD CSF profile, neuroinflammatory markers and the presence of NPS were tested by creating and interaction variable: pTau181/Aβ_1–42_ ratio × neuroinflammatory marker measurements. This was then used in a binary regression model with NPS occurrence as dependent variable. This approach tested whether a positive CSF biomarker profile for AD modified the effect of each individual neuroinflammatory on the occurrence of NPS.

### Association of neuroinflammation markers with progression of disease severity

NPI-Q scores at baseline were correlated with progression of disease severity as measured by changes in CDR-SoB at follow-up clinical assessments at 18 and 36 months in the whole cohort and in participants with a positive NPI-Q score using Spearman rho’s. Linear regression was used to test associations between CSF neuroinflammatory markers associated with NPS, and cognitive decline computed by change in CDR-SoB over time (CDR-SoB difference at follow-up/time between baseline and follow-up) corrected for baseline CDR-SoB assessment. Finally, we tested the association of the interaction between NPI-Q total score and sICAM-1 on CDR-SoB change using a binary logistic regression model using cognitive decline at 18 or 36 months (0 = no CDR-SoB change; 1 = CDR-SoB change) as dependent variable. We entered NPI-Q total score, sICAM-1 concentration and the interaction between NPI-Q score and sICAM-1 concentration (NPI-Q score × sICAM-1 levels) as variables into this model.

## Results

### Characteristics of the cohort

Demographic, clinical, and biological characteristics of participants with or without NPS (NPI-Q = 0 or NPI-Q > 0) are given in Table 1. The frequency of anxiety, apathy and depression measured by the NPI-Q is presented in Additional file 1: Table S1, by group with or without NPS as well as for individuals with CDR = 0 and NPI-Q > 0. Anxiety was the most common symptom in cognitively healthy participants. Longitudinal distribution of cognitive status (CDR = 0 or CDR > 0) and the occurrence of NPS within the cohort are shown in Additional file 1: Table S2. In both cases, larger changes in distribution were observed after 36 months. In addition, 67% of the participants with NPS at baseline presented NPS after 18 months (90% after 36 months) suggesting a persisting phenotype. In the whole cohort, total NPI-Q score was positively correlated with both CDR and CDR-SoB and negatively correlated with MMSE (data not shown).

**Table 1:** Demographic, clinical and biochemical characteristics of the study cohort

Clinical characteristic	NPI-Q = 0 (*n* = 48)	NPI-Q > 0 (*n* = 39)	*P* value
Sex, female, *n* (%)	34 (58.6)	24 (41.4)	0.360
Age, year, mean ± SD	68.19 ± 8.01	71.82 ± 6.1	0.022
Years of education ± SD	12.9 ± 2.3	12.1 ± 2.8	0.115
Cognitive function			
CDR, mean ± SD	0.2 ± 0.3	0.4 ± 0.3	< 0.001
CDR-SoB, mean ± SD	0.34 ± 1.03	1.71 ± 2.04	< 0.001
MMSE, ± SD	28.4 ± 1.92	26.03 ± 3.44	< 0.001
AD CSF biomarkers			
Aβ1–42, pg/ml, mean ± SD	939.72 ± 235.74	725.32 ± 257.72	< 0.001
Tau, pg/ml, mean ± SD	240.08 ± 113.76	507.56 ± 354.85	< 0.001
pTau181, pg/ml, mean ± SD	50.96 ± 21.87	75.32 ± 47.42	0.002
pTau/Aβ ratio, mean ± SD	0.06 ± 0.03	0.13 ± 0.12	< 0.001
Biochemical measures			
ApoEε4, *n* (%ε4)	8 (27.6)	21 (72.4)	< 0.001
QAlb, mean ± SD	5.02 ± 1.71	6.95 ± 2.62	< 0.001
Neuropsychiatric status			
NPI score, mean ± SD	0.0 ± 0.0	5.74 ± 5.26	< 0.001

### Neuroinflammatory signatures associated with the presence of NPS

The measured concentrations of all individual CSF and serum neuroinflammatory markers are shown in Additional file 1: Tables S3 and S4, respectively. We found 5 markers in CSF (IL-8, IL-15, sFLT-1, sICAM-1 and sVCAM-1, Additional file 1: Fig. S1A) and 1 in serum (MIP-1α, Additional file 1: Fig. S1B) displaying significantly different concentrations between participants with or without NPS. The concentrations of a similar set of molecules in CSF (sICAM-1, sVCAM-1, sFLT-1, IL-8 and IL-15, MCP-1 and MCP-4) showed positive correlations with total NPI-Q score in the whole cohort (Additional file 1: Fig. S1C). In serum, the concentration of MIP-1α showed a significant positive correlation with NPI-Q total score, while CRP and VEGF concentrations negatively correlated with NPI-Q total score (Additional file 1: Fig. S1D).

**Fig. 1:**
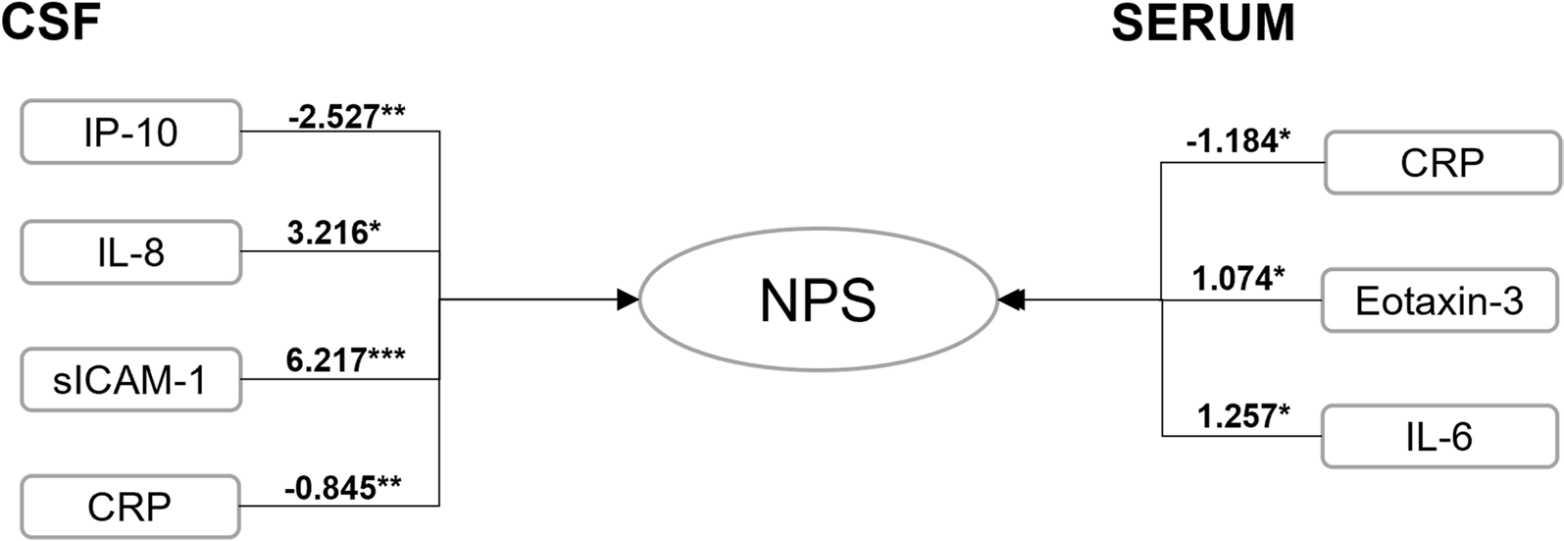
Binary logistic regression models revealing associations between neuroinflammatory molecules and neuropsychiatric symptoms (NPS). Associations of CSF (left) and serum (right) neuroinflammatory marker concentrations with the occurrence of NPS. Individual β-coefficients for each significantly associated neuroinflammatory molecule are shown. *P value < 0.05; **P value < 0.01; ***P value < 0.001. *CRP* C-reactive protein, *IP-10* 10-kDa IFN-γ induced protein, *sICAM-1* soluble intracellular adhesion molecule-1, *IL-8* Interleukin-8, *IL-6* Interleukin-6

Using binary regression models, we identified a combination of four neuroinflammatory markers in CSF that best predicted the occurrence of NPS: CRP, IP-10, sICAM-1 and IL-8 (Fig. 1). This CSF neuroinflammatory signature of NPS was unchanged when considering either baseline cognitive status or the presence of an AD CSF profile. The pTau181/Aβ_1–42_ ratio was also associated with NPS in this context (β-coefficient = 1.626 and *P*-value = 0.013). Each of the selected individual CSF neuroinflammatory markers was also associated with the volume of a specific subset of brain regions (Additional file 1: Table S5). In serum, the best predicting combination was: Eotaxin-3, IL-6 and CRP (Fig. 1). When considering baseline cognitive status only CRP (*β*-coefficient = − 0.752 and *P*-value = 0.042) remained associated with the presence of NPS; and both CRP (*β*-coefficient = − 0.784 and *P*-value = 0.033), and VEGF-D (*β*-coefficient = − 2.22 and *P*-value = 0.044) were associated with the presence of NPS when considering the CSF AD biomarker profile. In parallel, we also investigated the effects of BBB function on these associations by correcting our model for Qalb. In this model, the CSF inflammatory markers associated with the occurrence of NPS were CRP, IP-10 and sICAM-1. In serum, MIP-1α, VEGF were associated with the occurrence of NPS (data not shown). When added to a reference model built using *APOE* status, sex, age, years of education and cognitive status at baseline (Additional file 1: Table S6); the identified CSF signature of sICAM-1, IP-10, IL-8 and CRP together significantly contributed to improve prediction of NPS (Additional file 1: Fig. S2, AUC = 0.908,* P* value = 0.0058).

### Neuroinflammatory markers associated with single neuropsychiatric syndromes

**Fig. 2:**
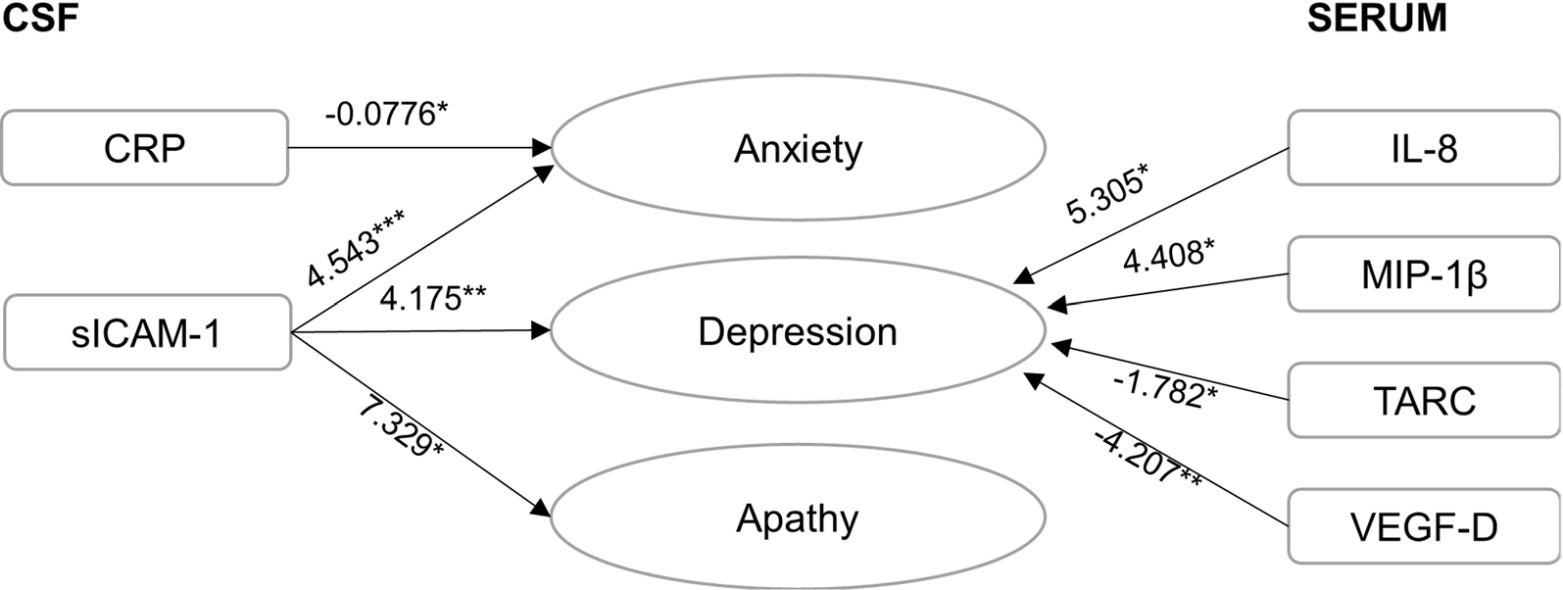
Associations of CSF (left) and serum (right) neuroinflammatory markers with anxiety, depression and apathy. Standardized *β*-coefficients obtained by regression models are shown. *P value < 0.05; **P value < 0.01; ***P value < 0.001. *CRP* C-reactive protein, *IP-10* 10-kDa IFN-γ induced protein, *sICAM-1* soluble intracellular adhesion molecule-1, *IL-8* Interleukin-8, *MIP-1β* macrophage inflammatory protein 1β, *TARC* thymus and activation-regulated chemokine, *VEGF-D*, vascular endothelial growth factor (D precursor)

We also identified specific CSF and serum neuroinflammatory markers associated with the occurrence of each depression, apathy and anxiety measured by NPI-Q (Fig. 2). In CSF, sICAM-1 was associated with the presence of all three symptoms and CRP was associated with the presence of anxiety only. In serum, we identified associations with the levels of IL-8, MIP-1β, TARC and VEGF-D, and the occurrence of depression.

### Interaction between neuroinflammation, NPS and AD pathology

Since the CSF concentrations of CRP, IP-10, sICAM-1, and a positive CSF biomarker profile for AD at baseline were associated with a positive NPI-Q score, we tested whether the interaction between neuroinflammation markers and a positive CSF biomarker profile for AD was also associated with NPS. Using binomial regression models with interaction variables (pTau181/Aβ_1–42_ ratio × individual neuroinflammatory markers), we found that only the interaction between the pTau181/Aβ_1–42_ ratio and CSF sICAM-1 was associated with the presence of NPS (Nagelkerke *R*^2^ = 0.363; coefficient = 3.386; *P* value = 0.027).

### NPS, neuroinflammation and progression of clinical disease severity

**Fig. 3:**
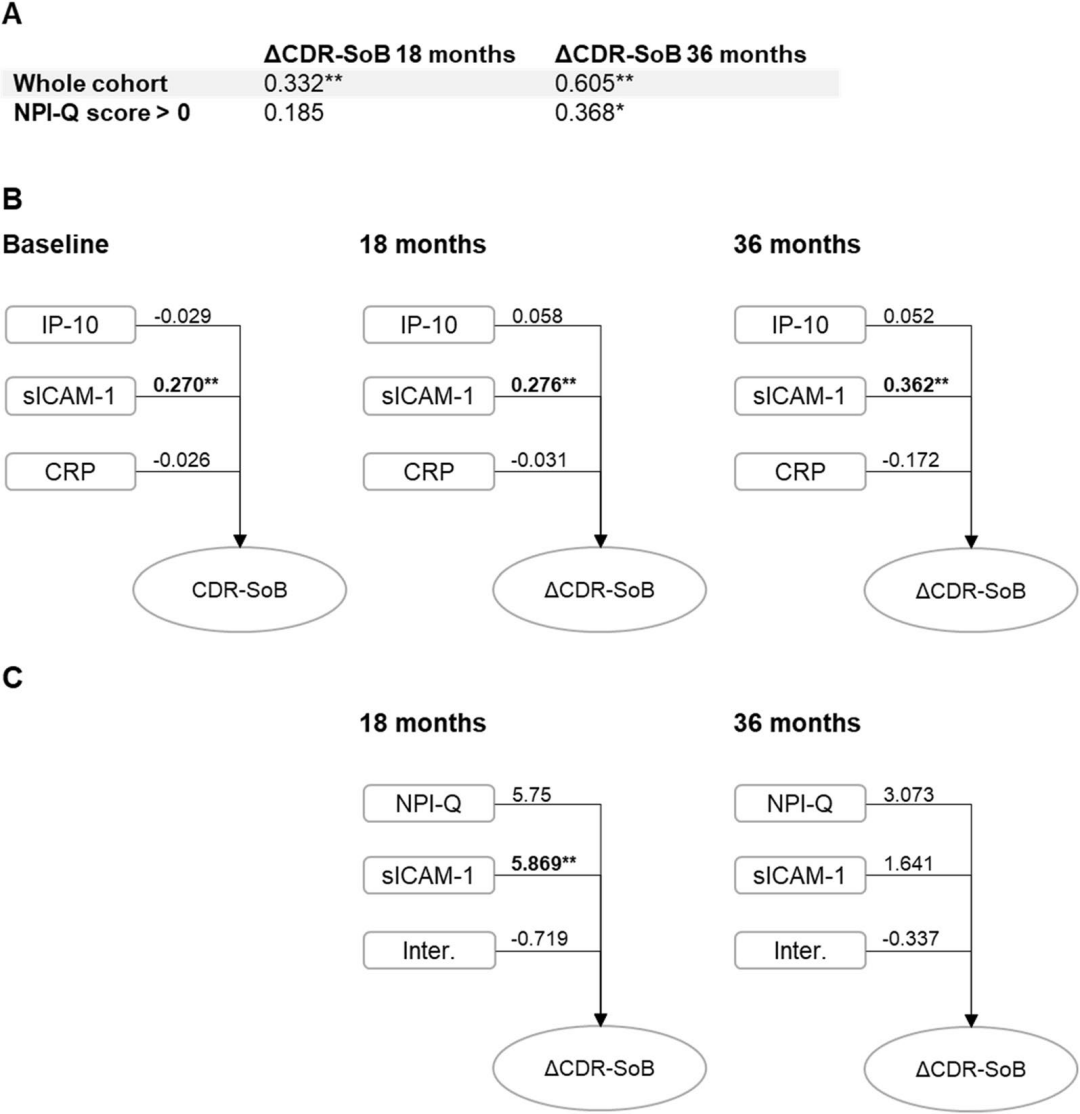
Associations of neuroinflammatory markers with progression of disease severity. **A** Correlations between NPI-Q total score at baseline and disease severity progression as measured by change in CDR-SoB (ΔCDR-SoB) over time. **B** Associations between neuroinflammatory markers and cognitive status at baseline and changes of disease severity in the whole cohort measured by change in CDR-SoB (ΔCDR-SoB) at 18 and 36 months. Standardized *β*-coefficients and obtained by linear regression models are shown. **C** Moderation model evaluating the contribution of NPI-Q score, CSF sICAM-1 at baseline and their interaction to disease severity progression at 18 and 36 months (ΔCDR-SoB). *β*-Coefficients obtained by binary logistic regression models are shown. CDR-SoB, Clinical Dementia Rating Sum of Boxes; CRP, C-reactive protein; sICAM-1, soluble intracellular adhesion molecule-1; IP-10, 10-kDa IFN-γ induced protein; NPI-Q, Neuropsychiatric Inventory Questionnaire score; Inter., Interaction Between NPI-Q score and sICAM-1 concentration (NPI-Q score × sICAM-1 levels)

NPI-Q at baseline was correlated with clinical severity progression as measured by changes in CDR-SoB at follow-up clinical assessments at 18 and 36 months (*n* = 87 and 85, respectively, Fig. 3A) in the whole cohort and in participants with a positive NPI-Q score. We also tested in a linear regression model the associations between the concentrations of molecules identified in the CSF signature in all models and disease severity progression. Only sICAM-1 was positively associated with disease severity progression at 18 and 36 months (Fig. 3B). A moderation model testing the interaction between NPI-Q score and sICAM-1 concentration revealed that the association between NPI-Q and CDR-SoB change at 18 months is affected by sICAM-1 concentration directly (Fig. 3C).

## Discussion

Assessing a large panel of neuroinflammation and vascular injury markers in paired blood and CSF samples we identified distinct systemic and CNS inflammatory signatures associated with the presence of neuropsychiatric symptoms, including altered levels of eotaxin-3, IL-6 and CRP in serum, and sICAM-1, IP-10, IL-8 and CRP in CSF. While NPS were more common in participants with cognitive impairment, the associations between CSF inflammation markers and NPS were independent of the presence of cognitive impairment at baseline. A significant interaction with AD pathology was observed for the association of NPS with CSF sICAM-1 only. Finally, the presence of NPS at baseline was associated with more rapid cognitive decline at follow-up visits, and this association was mediated by increased sICAM-1 in CSF.

While several CSF inflammation marker changes were associated with NPS, a combination of sICAM-1, IP-10, IL-8 and CRP levels best predicted the occurrence of NPS, with these molecules being independently associated with the NPS phenotype. This finding is in accordance with the concept that cytokines form a complex interplaying network enhancing and/or suppressing the production of each other, and together associating with clinical manifestation [31]. In the context of cognitive decline, sICAM-1 and CRP have previously been associated with AD pathology [19, 32]. IP-10 levels have been reported to be normal in AD [33], but have been associated with NPS and in particular with depression in one study [34]. CSF levels of IL-8 have previously been associated with both delirium and anxiety [35, 36]. A previous study has associated CRP levels with NPS [37]. CRP increases in the acute phase of immune response, but can also have a protective role by regulating inflammatory reactions via action on the complement system [38]. Taken together, the molecular signature of NPS we have identified is in line with most previous reports on single markers. The CSF signature of NPS was independent of the presence of cognitive impairment indicating that CNS neuroinflammation leading to NPS may also occur in the absence, or before the onset of cognitive decline. Furthermore, this signature was not dependent on BBB integrity, suggesting that the related inflammatory process originates within the CNS.

In serum, eotaxin-3, IL-6 and CRP levels together best predicted the presence of NPS. Very few previous studies have investigated, in older people in the context of cognitive decline or AD, associations of NPS with inflammation mediators from circulating blood. High serum concentrations of IL-6 and TNFα have been associated with the occurrence of depression and anxiety in patients with clinically diagnosed AD dementia [39]. Circulating CRP levels have also been associated with increased depression scores in an elderly non-demented population [40]. However, there is also conflicting evidence showing no association of CRP with depression in elderly people with AD-type dementia [39]. To our knowledge, no previous studies have linked serum eotaxin-3 levels with NPS. The serum inflammatory marker signature seems to depend on both BBB integrity and the presence of an AD CSF profile suggesting that both contribute to the association between systemic inflammation association and NPS. Previous studies have reported associations between inflammation mediators in circulating blood and atrophy in specific brain regions [41–43], suggesting neuroinflammation-driven neurodegeneration within these individual regions. A possible explanation may be that following BBB breakdown related to aging [44] or AD pathology [30], a cross-talk may occur between inflammation in the CNS and systemic inflammatory processes [45]. Systemic inflammation may contribute to CNS inflammation which may further result in cerebral dysfunction and neuronal injury, and the clinical manifestation of both cognitive impairment and NPS [18]. This was suggested for example for circulating IL-6 which may increase the concentration of sICAM-1 in the CNS, resulting in BBB damage and increased glial activation [46, 47]. On the other hand, systemic inflammation may also reflect cerebral pathology [21] and neuroinflammation related to core AD pathology [19].

This study extends previous evidence of associations between single CSF inflammatory molecules and depression and anxiety [35, 48–50] and reports evidence on apathy. Specifically, we observed a positive association between CSF sICAM-1 levels and the occurrence of anxiety, depression, and apathy. This suggests a role for this neuroinflammatory marker in the manifestation of these symptoms. Indeed, an association between sICAM-1 and depression has previously been reported [51]. On the other hand, some of the inflammatory molecules we investigated were specifically associated with a single symptom. In CSF, CRP was associated only with anxiety while in serum, IL-8, MIP-1α, TARC and VEGF-D were associated only with depression. This indicates distinct neuroinflammatory profiles for these syndromes. Some of these associations have previously been reported, such as serum IL-8 with anxiety [35]. Overall, the associations between neuroinflammation and individual syndromes observed in our study suggest that while in general neuroinflammation can result in a wide spectrum of neuropsychiatric manifestations, specific neuroinflammatory mediators could play a more important role in specific syndromes.

In the present study, the observed association between CSF neuroinflammatory markers and NPS could be driven by the large proportion of participants with AD pathology in the subgroup with NPS. However, when we accounted for the presence of cerebral core AD pathology in our models, the associations between CSF inflammatory markers and NPS was not altered. This indicates the CSF inflammatory signature of NPS is at least partially independent of AD. This is clinically relevant, as these inflammatory processes may cause NPS also in the absence of AD pathology, and therefore may be considered a distinct pathophysiological aspect from AD.

Baseline NPI-Q score was correlated with progression in the clinical severity at follow-up visits, in line with previous observations [2, 5, 7]. Also, higher CSF sICAM-1 concentration was associated with more rapid progression of disease severity, i.e., more rapid cognitive and related functional decline. Furthermore, the interaction analysis showed that the association between the presence of NPS at baseline and clinical disease progression is mediated by CSF sICAM-1 levels. Together with the observed CSF sICAM-1 in associations with all single neuropsychiatric syndromes, this finding suggests a key role of sICAM-1 for the pathogenesis of NPS and the association of NPS with clinical disease progression.

**Fig. 4:**
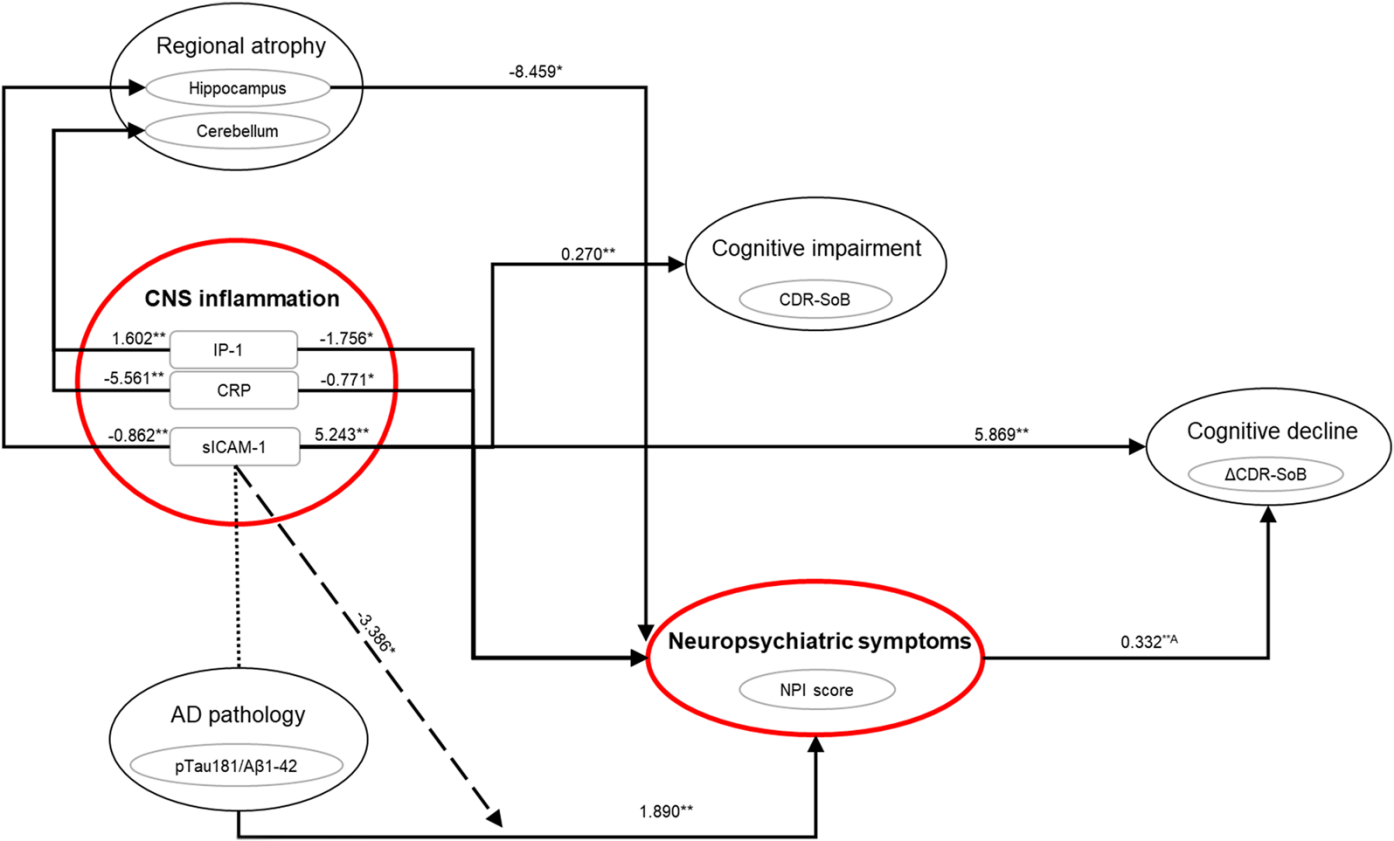
Graphical representation of the role of CNS neuroinflammation, in NPS, functional cognitive decline and regional brain atrophy. Significant associations or interactions are represented by arrows with standardized coefficients indicated alongside. The dashed arrow represents the interaction of sICAM-1 on the association between AD pathology and NPS. The dotted line represents an association demonstrated in a previous study [19]. Associations between neuroinflammation and NPS are described in detail in Fig. 1 and are independent of BBB function and cognitive status. *P value < 0.05; **P value < 0.01; ***P value < 0.001; C-reactive protein; IP-10, 10-kDa IFN-γ induced protein; sICAM-1, soluble intracellular adhesion molecule-1; *A*, correlation coefficient

sICAM-1 was further associated with the volume of the hippocampus and 3rd ventricle (reflecting atrophy of surrounding areas). These regions have been associated with both neurodegeneration in AD and inflammation in previous works [52, 53]. In addition, CSF sICAM-1 was previously found to correlate with measures of both neurodegeneration and tau hyperphosphorylation in the CSF [19]. Together, this suggests that regional neurodegeneration and related neuroinflammation with increased sICAM-1 levels represent underlying pathology of the occurring NPS, in particular of anxiety, depression and apathy. Considering previous studies showing that brain regions associated with NPS [54] are also associated with more rapid disease progression in AD [55], our findings suggest that the interaction between sICAM-1 driven neuroinflammation and AD pathology may occur within the hippocampus and the areas surrounding the 3rd ventricle. Therefore, increased sICAM-1 related inflammation in the CNS appears to be both involved in neuronal injury and tau pathology and contribute to NPS, and in particular in the context of AD (Fig. 4).

This is to our knowledge the first study to assess a large panel of inflammatory markers in paired serum and CSF samples to determine inflammatory signatures of NPS in older people while considering cognitive status and biomarkers of AD pathology. The size of our cohort does not allow for stratification into subgroups according to the A/T/(N) classification framework, limiting further exploration of the relationship between the identified neuroinflammatory marker profiles and AD pathology and how neuroinflammation could play differential roles in different pathological presentations. A further limitation of this study is related to the fact that subjects included in this cohort had no psychiatric disorder or severe psychiatric symptoms that could interfere with cognition, and that candidates with more marked neuropsychiatric symptoms were not considered. However, both elderly subjects with normal cognition and with early stages of cognitive decline were considered, allowing to evaluate the relevance for clinical disease progression over time. Apathy had a relatively low frequency in our cohort and some relationships with this syndrome could have been missed. Furthermore, we did not include subjects with more advanced stages of dementia, where the pathogenesis of NPS may differ from early stages of cognitive decline. Whether the identified signatures can be used in clinical practice as markers of neuroinflammation-related NPS and as targets for intervention needs to be further investigated in independent samples.

## Conclusions

We have identified specific CSF and serum inflammatory signatures associated with NPS that indicate distinct contributions of systemic and CNS inflammation to the pathophysiology of NPS. This study is the first to relate inflammation markers to NPS in older people with normal cognition or with cognitive impairment while considering AD pathology as indicated by CSF biomarkers. While the CSF signature appears to indicate inflammatory processes that originate within the CNS and are independent of the core AD pathology, the serum signature may represent systemic inflammatory activity which may contribute to pathological processes in the CNS that clinically manifest as NPS. The inflammatory signatures of NPS indicate syndrome-specific underlying processes opening the perspective of targeted interventions to reduce NPS and their long-term consequences.

## Supplementary Information

**Additional file 1: Table S1.** Neuropsychiatric symptom distribution within the study cohort. **Table S2.** Longitudinal distribution of cognitive and neuropsychiatric status within the cohort. **Table S3.** Concentrations (in pg/ml) of neuroinflammatory markers in CSF. **Table S4.** Concentrations (in pg/ml) of neuroinflammatory markers in serum. **Table S5.** Associations of volumetric data with neuroinflammatory marker concentration. **Table S6.** Reference model. **Figure S1.** Concentrations and correlations of neuroinflammatory markers with NPI-Q score in the whole cohort. **Figure S2.** Predictive model of the presence of neuropsychiatric symptoms.

## Data Availability

The datasets used and/or analyzed during the current study are available from the corresponding author on reasonable request.

## Abbreviations

Aβ1–42: Beta-amyloid 1–42
AD: Alzheimer’s disease
AUC: Area under the curve
BBB: Blood–brain barrier
CDR: Clinical Dementia Rating
CDR-SoB: CDR Sum of Boxes
CRP: C-reactive protein
CSF: Cerebrospinal fluid
CNS: Central nervous system
IFN-γ: Interferon-γ
IL: Interleukin
IP-10: 10-KDa interferon-γ-induced protein
MCP-1,4: Monocyte chemoattractant protein 1,4
MDC: Macrophage-derived chemokine
MIP-1α: Macrophage inflammatory protein 1α
MMSE: Mini-Mental State Examination
NPI-Q: Neuropsychiatric Inventory Questionnaire
NPS: Neuropsychiatric symptoms
pTau181: Tau phosphorylated at threonine 181
Qalb: CSF albumin index
ROC: Receiver operating characteristic
SAA: Serum amyloid A
sFLT-1: Soluble fms-like tyrosine kinase-1
sICAM-1: Soluble intracellular cell adhesion molecule-1
TARC: Thymus and activation-regulated chemokine
Tau: Total-tau
TIE-2: Angiopoietin-1 receptor
VEGF: Vascular endothelial growth factor
VEGF-D: Vascular endothelial growth factor D precursor
VIF: Variance inflation factor
