# Supplementary material for: Systemic and central nervous system neuroinflammatory signatures of neuropsychiatric symptoms and related cognitive decline in older people
Source: J Neuroinflammation. 2022 May 28;19:127. doi: 10.1186/s12974-022-02473-3 (PMC9148517; doi:10.1186/s12974-022-02473-3)
Supplement: Supplementary file 1 — Additional file 1: Table S1. Neuropsychiatric symptom distribution within the study cohort. Table S2. Longitudinal distribution of cognitive and neuropsychiatric status within the cohort. Table S3. Concentrations (in pg/ml) of neuroinflammatory markers in CSF. Table S4. Concentrations (in pg/ml) of neuroinflammatory markers in serum. Table S5. Associations of volumetric data with neuroinflammatory marker concentration. Table S6. Reference model. Figure S1. Concentrations and correlations of neuroinflammatory markers with NPI-Q score in the whole cohort. Figure S2. Predictive model of the presence of neuropsychiatric symptoms. [file 12974_2022_2473_MOESM1_ESM.docx]

Systemic and central nervous system neuroinflammatory signatures of neuropsychiatric symptoms and related cognitive decline in older people

Christopher Clark, PhD^a^, Jonas Richiardi, PhD^b^, Bénédicte Maréchal, PhD^c^, Gene L. Bowman MD, PhD^d^, Loïc Dayon, PhD^d,e,f^ and Julius Popp, MD,PhD^a,g,h^

a: Institute for Regenerative Medicine, University of Zürich, Wagistrasse 12, 8952 Schlieren, Switzerland; christopher.clark@irem.uzh.ch

b: Department of Radiology, Lausanne University Hospital and University of Lausanne, CHUV CH-1011  Lausanne, Switzerland; jonas.richiardi@chuv.ch

c: Advanced Clinical Imaging Technologies group, Siemens Healthcare Switzerland, CH-1015 Lausanne, Switzerland; benedicte.marechal@siemens-healthineers.com

d: Nestlé Institute of Health Sciences, Nestlé Research, EPFL Innovation Park, Bâtiment H, CH-1015 Lausanne, Switzerland; drgenebowman@gmail.com,

e: Nestlé Institute of Food Safety & Analytical Sciences, Nestlé Research, Lausanne, Switzerland; loic.dayon@rd.nestle.com

f: Institut des Sciences et Ingénierie Chimiques, Ecole Polytechnique Fédérale de Lausanne, Lausanne, Switzerland

g: Centre Hospitalier Universitaire Vaudois, Rue du Bugnon 46, 1011 Lausanne, Switzerland ;

h: University Hospital of Psychiatry Zürich, Department of Geriatric Psychiatry, Centre for Gerontopsychiatric Medicine, Minervastrasse 145, P.O. Box 341 8032 Zürich, Switzerland; julius.popp@puk.zh.ch

Corresponding Authors:

Christopher Clark

Institute for Regenerative Medicine, University of Zürich, Wagistrasse 12, 8952 Schlieren, Switzerland; [christopher.clark@irem.uzh.ch](mailto:christopher.clark@irem.uzh.ch)

Julius Popp

Centre for Gerontopsychiatric Medicine, University Hospital of Psychiatry Zürich, Department of Geriatric Psychiatry, Minervastrasse 145, P.O. Box 341 8032 Zürich, Switzerland; [julius.popp@pukzh.ch](mailto:julius.popp@pukzh.ch)

**Supplementary Methods:**

*Group comparisons:*

We used box plots and t-tests to compare biomarker distribution and concentration between participants with or without NPS. Correlations between NPI-Q total score and neuroinflammatory markers were assessed with Spearman’s rho. Benjamini-Hochberg correction of *P* value for multiple testing was then applied using a false-discovery rate of 0.25. Considering our effect sizes (>0.7), the size of this cohort results in a statistical power of 0.9 or more.

*Regional brain volumetric measurements:*

All participants underwent a magnetic resonance imaging scan on a 3T MRI system (MAGNETOM Prisma^fit^, Siemens Healthcare, Erlangen, Germany) with a 32-channel head coil. Acquisitions followed the ADNI2 MRI protocol ^1^. Images were then segmented with the MorphoBox prototype algorithm ^2^; briefly, this registers subject data to an internal template established by consensus segmentation of neuroradiologists, applies bias field correction with a 4-tissue class (gray matter (GM), white matter (WM), cerebro-spinal fluid (CSF), non-brain) Gaussian mixture model, performs skull-stripping, classifies brain tissue into 5 classes (ventricular CSF, sulcal CSF, cortical GM, deep GM, and WM) via variational expectation-maximisation yielding 5 posterior probability maps, and provides regional volume estimates by summing up these probabilities within template regions. Quality was checked using established automated image quality ^3^ and segmentation quality metrics ^2^, and no images were rejected. We analyzed 18 brain structures and features including: amygdala, caudate nucleus, cerebellum, cortical grey matter, grey matter, hippocampus, insula, medulla oblongata, mesencephalon, pallidum, pons, putamen, thalamus, 3^rd^ ventricle, 4^th^ ventricle, total ventricular volume, white matter, white matter abnormalities. This regional volumetric data was normalized by total intracranial volume (defined as the sum of gray matter, white matter and CSF).

*Association of neuroinflammatory markers and brain volumetric measurements.*

To assess if the association between NPS and selected CSF neuroinflammatory molecules was mediated by or occurring in specific brain regions, we used linear regression models with stepwise selection method and selected individual neuroinflammatory CSF molecule concentration as dependent variables entering all volumetric measurements.

*Evaluation of the diagnostic performance of the selected neuroinflammatory signatures*

A reference model was constructed using binary logistic regression with NPI-Q>0 or NPI-Q=0 as dependent variable while using only demographic and clinical measures, including *APOEε4* status, sex, age, years of study and cognitive status in order to predict the occurrence of NPS. A receiver operating characteristic (ROC) curve and area under the curve (AUC) were computed for this reference model. CSF markers associated with the occurrence of NPS selected by our binomial logistic regression models where then added to this model in a consecutive block and ROC curves and AUCs were compared using the DeLong method.**Supplementary Tables:**

|  | NPI-Q = 0 n (%) | NPI-Q >0, n (%) | CDR = 0, n (%) |
| --- | --- | --- | --- |
| Total NPI-Q score | 48 (55.2) | 39 (44.8) | 11 (12.6) |
| Depression | 70 (82.4) | 15 (17.6) | 5 (5.7) |
| Anxiety | 60 (70.6) | 25 (29.4) | 7 (8) |
| Apathy | 81 (95.3) | 4 (4.7) | 2 (2.3) |

**Supplementary Table 1:** Distribution of individual NPS within the study cohort. CDR = 0 indicate participants with no cognitive impairment but presenting NPS.

| Cognitive status | CDR = 0, n (%) | CDR > 0, n (%) |
| --- | --- | --- |
| *Baseline* | 42 (48,3) | 45 (51,7) |
| *18 months follow-up* | 40 (50,6) | 39 (49,4) |
| *36 months follow-up* | 30 (44,1) | 38 (55,9) |
|  |  |  |
| Neuropsychiatric status | **NPI-Q = 0, n (%)** | **NPI-Q > 0, n (%)** |
| *Baseline* | 48 (55,2) | 39 (44,8) |
| *18 months follow-up* | 39 (50,6) | 38 (49,4) |
| *36 months follow-up* | 19 (38,8) | 30 (61,2) |

**Supplementary Table 2:** Longitudinal distribution of cognitive (top) and neuropsychiatric (bottom) status within the cohort. CDR, Clinical Dementia Rating; NPI-Q, Neuropsychiatric inventory questionnaire score.

| Neuroinflammatory marker | NPI = 0 (n = 48) | NPI > 0 (n = 38) | *P* value^*^ |
| --- | --- | --- | --- |
| C-reactive protein | 5650.71 ± 5887.25 | 4016.05 ± 4200.64 | 0.142 |
| Interleukin-12 | 5.28 ± 1.52 | 5.66 ± 1.71 | 0.294 |
| Interleukin-15 | 3.51 ± 0.90 | 3.94 ± 0.89 | **0.032** |
| Interleukin-16 | 12.61 ± 3.25 | 12.92 ± 3.19 | 0.659 |
| Interleukin-6 | 1.08 ± 0.39 | 1.06 ± 0.40 | 0.812 |
| Interleukin-7 | 1.36 ± 0.55 | 1.41 ± 0.64 | 0.680 |
| Interleukin-8 | 38.70 ± 8.40 | 42.36 ± 8.21 | **0.048** |
| 10 kDa IFN-γ induced protein | 570.94 ± 225.50 | 551.88 ± 213.14 | 0.700 |
| Monocyte chemoattractant protein 1 | 425.07 ± 103.07 | 460.09 ± 120.54 | 0.156 |
| Monocyte chemoattractant protein 4 | 4.96 ± 3.59 | 6.20 ± 3.22 | 0.101 |
| Macrophage-derived chemokine | 33.48 ± 16.82 | 30.37 ± 20.16 | 0.450 |
| Macrophage Inflammatory protein 1α | 19.42 ± 6.40 | 20.27 ± 6.72 | 0.556 |
| Macrophage Inflammatory protein 1β | 14.18 ± 3.26 | 14.56 ± 3.41 | 0.597 |
| Phosphorylated insulin-like growth factor-1 receptor | 75.04 ± 27.63 | 82.39 ± 26.59 | 0.220 |
| Serum amyloid A | 999.41 ± 670.95 | 1256.43 ± 1004.76 | 0.168 |
| Soluble fms-like tyrosine kinase-1 | 52.76 ± 13.00 | 62.83 ± 16.64 | **0.004** |
| Soluble intercellular adhesion molecule-1 | 2077.59 ± 513.74 | 2708.04 ± 637.78 | **<0.001** |
| Circulating vascular cell adhesion molecule-1 | 7416.46 ± 1983.03 | 9082.72 ± 2726.64 | **0.002** |
| Thymus and activation-regulated chemokine | 4.10 ± 2.23 | 3.71 ± 1.96 | 0.403 |
| Vascular endothelial growth factor | 4.08 ± 0.71 | 4.14 ± 0.96 | 0.774 |
| Vascular endothelial growth factor D precursor | 40.29 ± 13.90 | 47.44 ± 18.52 | 0.052 |

**Supplementary Table 3:** Concentrations (in pg/mL) of neuroinflammatory markers in CSF. ^*^*P* value represents result of t-test comparing NPI and control group for continuous variables with Levene’s test for equality of variances. Significant *P* values after multiple comparison correction are highlighted in bold.

| Neuroinflammatory marker | NPI = 0 (n = 47) | NPI > 0 (n = 39) | *P* value* | |
| --- | --- | --- | --- | --- |
| Basic fibroblast growth factor | 3.61 ± 2.29 | 3.65 ± 2.31 | 0.948 | |
| C-reactive protein | 2212886.73 ± 2101109.56 | 1303176.49 ± 1509462.94 | 0.024 |  |
| Eotaxin-1 | 146.58 ± 48.92 | 170.00 ± 44.00 | 0.065 |  |
| Eotaxin-3 | 30.38 ± 16.77 | 39.37 ± 15.89 | 0.041 |  |
| IFN-γ | 8.11 ± 5.74 | 7.83 ± 5.01 | 0.821 |  |
| Interleukin-12 | 148.22 ± 63.12 | 165.85 ± 73.75 | 0.238 |  |
| Interleukin-15 | 2.88 ± 0.44 | 3.01 ± 0.51 | 0.204 |  |
| Interleukin-16 | 205.97 ± 53.92 | 222.97 ± 52.39 | 0.147 |  |
| Interleukin-6 | 0.67 ± 0.33 | 0.71 ± 0.24 | 0.459 |  |
| Interleukin-7 | 29.26 ± 7.81 | 29.16 ± 7.94 | 0.952 |  |
| Interleukin-8 | 14.13 ± 4.02 | 13.42 ± 4.58 | 0.171 |  |
| 10 kDa IFN-γ induced protein | 340.28 ± 143.64 | 354.76 ± 129.20 | 0.692 |  |
| Monocyte chemoattractant protein 1 | 314.90 ± 87.72 | 335.33 ± 99.70 | 0.403 |  |
| Monocyte chemoattractant protein 4 | 168.52 ± 70.92 | 191.95 ± 70.75 | 0.210 |  |
| Macrophage-derived chemokine | 1072.52 ± 317.41 | 1013.39 ± 345.78 | 0.488 |  |
| Macrophage Inflammatory protein 1α | 20.61 ± 8.50 | 26.04 ± 6.09 | **0.005** |  |
| Macrophage Inflammatory protein 1β | 99.32 ± 32.07 | 99.62 ± 37.09 | 0.970 |  |
| Phosphorylated insulin-like growth factor-1 receptor | 27.21 ± 6.23 | 27.71 ± 7.31 | 0.733 |  |
| Serum amyloid A | 5410242.82 ± 4167963.83 | 3932000.33 ± 3237582.71 | 0.076 |  |
| Soluble fms-like tyrosine kinase-1 | 105.41 ± 26.03 | 115.00 ± 31.41 | 0.132 |  |
| Soluble intercellular adhesion molecule-1 | 390796.15 ± 83047.90 | 356076.83 ± 79787.33 | 0.055 |  |
| Circulating vascular cell adhesion molecule-1 | 596622.52 ± 131559.39 | 561172.03 ± 114880.69 | 0.191 |  |
| Thymus and activation-regulated chemokine | 277.08 ± 139.05 | 242.68 ± 160.33 | 0.380 |  |
| Angiopoietin-1 receptor | 6390.58 ± 1380.07 | 6145.48 ± 1542.84 | 0.442 |  |
| TNF-α | 2.7624 ± 0.81 | 2.85 ± 0.68 | 0.599 |  |
| Vascular endothelial growth factor | 143.98 ± 86.40 | 117.32 ± 95.45 | 0.188 |  |
| Vascular endothelial growth factor C | 503.47 ± 170.55 | 511.64 ± 169.11 | 0.825 |  |
| Vascular endothelial growth factor D precursor | 801.17 ± 207.35 | 763.43 ± 224.53 | 0.424 |  |

**Supplementary Table 4:** Concentrations (in pg/mL) of neuroinflammatory markers in serum. ^*^*P* value represents result of t-test comparing NPI and control group for continuous variables with Levene’s test for equality of variances. Significant *P* values after multiple comparison correction are highlighted in bold.

| ***Marker and associated regions*** | ***R^2^*** | ***Coeff.*** |
| --- | --- | --- |
| **C-reactive protein** | 0.146 |  |
| Cerebellum |  | -5.561^**^ |
| Pons |  | 3.106^*^ |
| **10 kDa IFN-γ induced protein** | 0.233 |  |
| Insula |  | -1.598^***^ |
| Cerebellum |  | 1.602^**^ |
| Pallidum |  | 1.110^*^ |
| **Soluble intracellular adhesion molecule-1** | 0.307 |  |
| Hippocampus |  | -0.862^**^ |
| 3^rd^ Ventricle |  | 0.362^**^ |

**Supplementary Table 5:** Associations of volumetric data with cerebrospinal fluid neuroinflammatory marker concentration assessed with linear regression models. Individual model R^2^ as well as β-coefficients for each significantly associated neuroinflammatory molecule are shown. * *P* value < .05; ** *P* value < .01; *** *P* value < .001.

| Nagelkerke R^2^ = 0.335 | AUC = 0.808 |  |
| --- | --- | --- |
| Factor: | **Coeff.** | **P value** |
| *APOE4* status | 1.833 | 0.002 |
| Gender | -0.460 | 0.399 |
| Age | 0.18 | 0.654 |
| Years of education | -0.218 | 0.065 |
| CDR > 0 | 0.931 | 0.114 |

**Supplementary Table 6:** Reference model. Associations of clinical and demographic characteristics with neuropsychiatric symptoms. Results from binary logistic regression analysis are presented with β-coefficient for each factor in the model. *P* value is derived from Wald statistics. Note that only *APOE* status is significantly associated with the occurrence of neuropsychiatric symptoms. AUC: area under ROC curve.

**Supplementary Figures:**


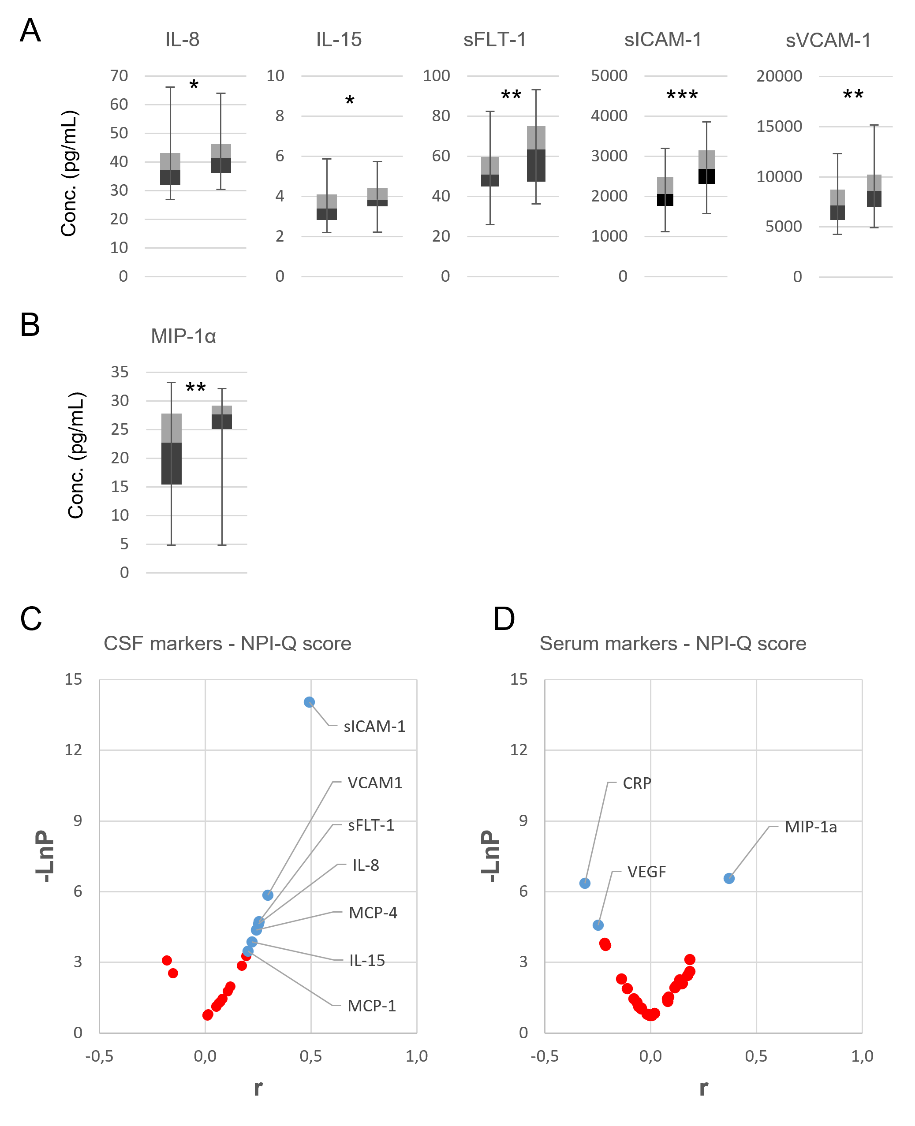


**Supplementary Figure 1:** Concentrations and correlations of neuroinflammatory markers in cerebrospinal fluid (CSF) and serum with Neuropsychiatric inventory questionnaire (NPI-Q) score in whole cohort. A-B) Box plots of molecules showing a significantly different concentration in CSF (A) and serum (B) between subjects without(left) and participants with neuropsychiatric symptoms (right). C-D) Correlation of CSF(C) and serum (D) markers with NPI-Q score. Significantly correlated markers after correction for multiple testing are shown in blue and labelled on the graph. IL-8, Interleukin-8; IL-15, Interleukin-15; sFLT-1, soluble fms-like tyrosine kinase-1; sICAM-1, soluble intracellular adhesion molecule-1; sVCAM-1, circulating vascular cell adhesion molecule-1; MIP-1α, macrophage Inflammatory protein 1α; CRP, C-reactive protein; MCP-1(4), monocyte chemoattractant protein 1(4); VEGF, vascular endothelial growth factor; * *P* value < .05; ** *P* value < .01; *** *P* value < .001; –LnP, inverse logarithm of *P* value; r Spearman’s rho.


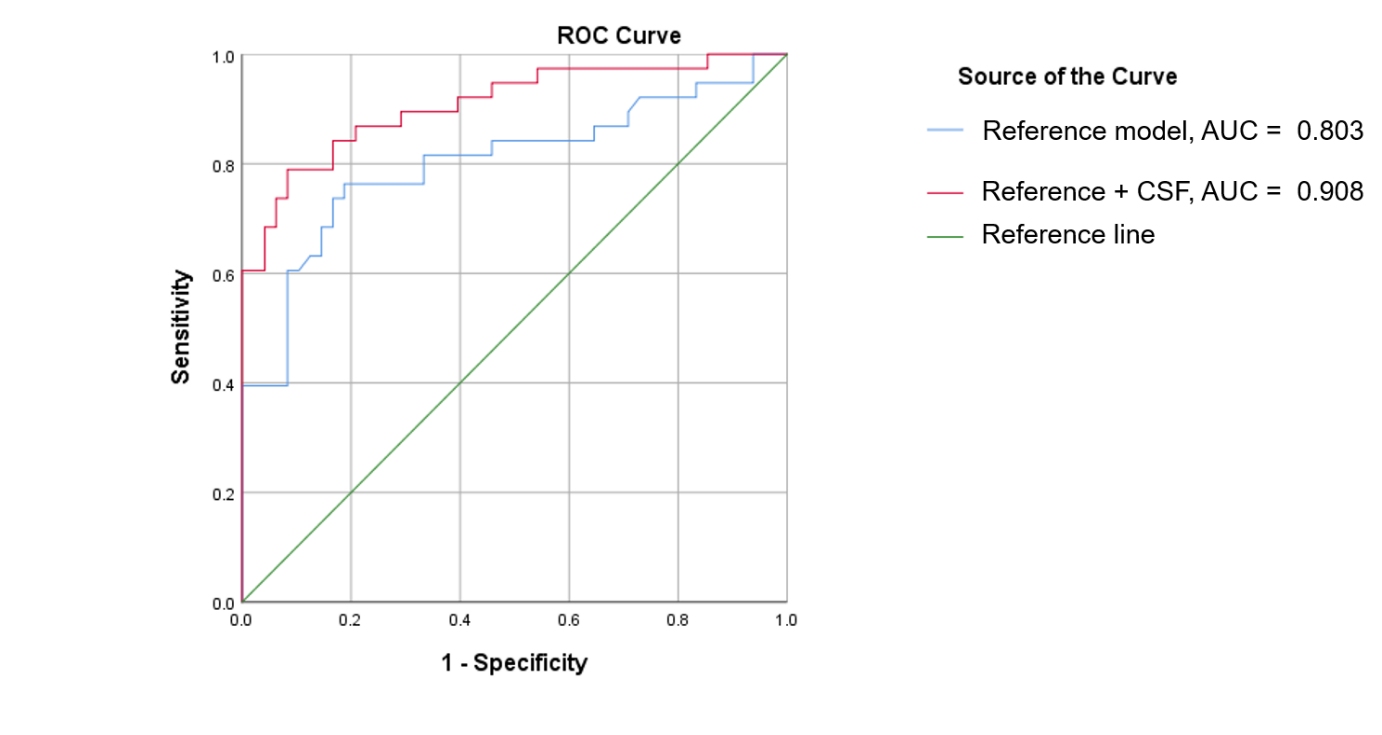


**Supplementary Figure 2:** Predictive model of the presence of neuropsychiatric symptoms. Probabilities derived from a binary logistic regression model using demographic and clinical factors as a primary block (reference model, blue) and four neuroinflammatory cerebrospinal fluid markers (sICAM-1, IP-10, IL-8 and CRP) as a secondary block (red) shown as a ROC curve. AUC = Area under the Curve.

References

1. ADNI Study Documents. Available at http://adni.loni.usc.edu/methods/documents/, accessed Accessed 27 Apr 2020.

2. Schmitter D. *et al.* An evaluation of volume-based morphometry for prediction of mild cognitive impairment and Alzheimer's disease. *NeuroImage. Clinical* **7**, 7–17 (2015).

3. Mortamet B. *et al.* Automatic quality assessment in structural brain magnetic resonance imaging. *Magnetic resonance in medicine* **62**, 365–372 (2009).
